# Work-related musculoskeletal disorders and associated factors among weavers working in Bahir Dar City, Northwest Ethiopia: cross-sectional study design

**DOI:** 10.1186/s12891-024-07494-y

**Published:** 2024-05-29

**Authors:** Fiseha Sefiwu Zinabu, Kefale Getie, Kassaw Belay Shiferaw, Gashaw Jember Belay, Mihret Dejen Takele, Molla Fentanew, Belaynew Addis Mekuria, Bewunetu Fenta Getaneh, Yeshambel Ejigu Anteneh, Alemu Kassaw Kibret

**Affiliations:** 1 Department of Physiotherapy, School of Medicine, College of Medicine and Health Sciences, University of Gondar, P.O. Box 196, Gondar, Ethiopia; 2 Department of Physiotherapy, School of Medicine, College of Medicine and Health Sciences, Bahir Dar University, Bahir Dar, Ethiopia

**Keywords:** Prevalence, Associated factors, Work-related musculoskeletal disorder, And weavers

## Abstract

**Background:**

Work-related musculoskeletal disorders are one of the most common public health problems throughout the world. It is a major cause of occupational injury, illness, and disability that leads to an increased rate of sick leave, financial costs for both individuals and society and the health care system. Weavers are among the populations exposed to musculoskeletal disorders due to a lack of occupational safety, health services, and poor working conditions. Yet, little is known about WMSD among weavers in Africa particularly in Ethiopia. Thus, this study aimed to assess the prevalence and associated factors of work-related musculoskeletal disorders among weavers.

**Method:**

A multi-centered institutional-based cross-sectional study was conducted in Bahir Dar City, Ethiopia among 424 weavers with a response rate of 97.7%. Participants were recruited randomly after proportional allocation was done for each institution and face-to-face interviews were employed to collect data using a well-structured questionnaire adopted from the Nordic musculoskeletal questionnaire and different literature. The data were entered into Epidata and analyzed using SPSS version (Choobineh A, Lahmi M, Shahnavaz H, Khani Jazani R, Hosseini M. Int J Occup Saf Ergon, 10:157–68, 2004). Variables with a P-value < 0.25 in the bivariate logistic regression were entered into a multivariable logistic regression model. Finally, variables with an AOR, a 95% confidence interval, and a P-value of < 0.05 were reported.

**Result:**

The annual prevalence rate of work-related musculoskeletal disorders among weavers was 76.3% [CI = 72.2 to 80.2%]. Longer working hours [AOR = 3.48, CI = (1.96–6.21)] lack of back support during sitting [AOR = 2.50 (1.293–4.480)], having repetitive movement in weaving [AOR = 4.11(2.029–8.324)], attaining awkward posture [AOR = 3.37(1.875–6.059)] and job stress [AOR = 4.37(2.447–7.816)] was significantly associated with work-related musculoskeletal disorders.

**Conclusions:**

This study revealed a high prevalence of WMSDs among weavers. Our study findings reported that longer working hours, lack of back support during sitting, having repetitive movement in weaving attaining awkward posture, and having job stress were significantly associated with work-related musculoskeletal disorders.

## Background

Musculoskeletal disorder (MSD) is described as a problem of the muscular system which includes muscles, nerves, tendons, ligaments, joints, and cartilageas well as the supporting structure of neck, back, and affects any area of the body [1, 2]. Work-related musculoskeletal disorders (WMSDs) are currently one of the most serious health problems that ergonomists face in the workplace all around the world [3]. It is a significant threat to the health of employees in both developed and developing countries [4]. In a previous study on global burden disease in 2010, WMSD was the fourth biggest burden on the health of the world’s population. It is estimated that WMSD accounts for approximately 6.7% of global disability-adjusted life years [5, 6].

WMSDs are a major cause of morbidity in many countries and have emerged as the leading cause of occupational injury, illness, and disability. It increased rates of sick leave and enormous financial costs for both individuals and society which ultimately have a detrimental effect on work productivity and business continuity [7–9]. WMSD also creates a burden on the health system, economic, and social costs [10].

WMSD worsens or continues for longer than anticipated due to job or task performance, workplace circumstances, and working conditions [11]. The healthcare sector, transportation, mining, food regulation, leather tanning, and manufacturing are all areas of greatest risks [12]. Weavers are among the populations at risk for WMSDs due to their long working hours and strenuous activities [13]. It is considered to be one of the world’s oldest surviving crafts [14]. Weaving is an important cottage industry in developed and developing countries including India, Pakistan, Bangladesh, Iran, Turkey, and China, where traditional weaving methods are still widely used  [13]. Every year, over 1.5 million weavers, dyers, hand spinners, embroiderers, and other employees utilize over 0.3 million operating looms to produce 620 million metric tons of cloth [15]. A handloom is a machine or tool constructed of wood and iron that is used to weave cloth without the use of electric motors; instead, weavers move the fabric with their hands and feet [16, 17].

Weaving involves a variety of tasks that are done by constantly sitting in a static position to weave fabric and through repeated movement of upper and lower limbs to operate pedals and shuttles with arms extended away from the body [18]. Weavers who do not take exercise regularly and illiterate are at high risk of musculoskeletal pain [19]. Weaving activities demand a repetitive effort that strains the musculoskeletal system, increasing the chance of exhaustion and reducing the opportunity for tissue to recover, resulting in pain and discomfort [20]. Several studies on the weaving sector found substantial WMSDs related to jobs [7, 14, 21, 22].

WMSDs lead wavers to different levels of physical disabilities [23], taking medication for their pain [24], preventing activities of daily living, and missing work due to WMSD [25, 26]. Previous evidence showed that age, sex, BMI (body mass index), and educational background are factors associated with WMSDs [13, 27–29]. WMSDs are also significantly associated with the absence of breaks, working experience, awkward work postural demand, working days, working more than eight hours per day, Working seat without a back support, repetitive movement, and working in the static position [13, 23, 30–33]. Behavioral and Psychological factors are also associated with WMSD such as alcohol, smoking, physical inactivity, job dissatisfaction job stress [13, 30, 34–36]. The prevalence of WMSD is variably reported from different studies in the last 12 months, as an instance in India was 100%, four studies in Bangladesh was 100%, 82.4% 80.5%, and 27.4% [13, 19, 32, 37, 38]. Evidence on the management and prevention of musculoskeletal disorders emphasized the importance of self-management, regular physical exercise,  workplace engineering redesign, good working posture, administrative controls , ergonomic training, protective equipment, and medical management are recommended.  In addition to this pain management, exercise therapy, manual therapy, and acupuncture are also recommended [39–41].

Despite WMSDs among weavers being the most common public health issue that hurts function, performance, and productivity.  There is limited evidence that shows the magnitude and associated factors of WMSD among weavers in Africa particularly in the study area, Ethiopia. Information provided by this study can be used to emphasize the need for primary prevention of WMSD by promoting health with the weaver’s personnel and the economic development of the country. Further more, the findings of this study are also significant in determining the extent of the problem,  identifying risk factors,  prevention strategies to reduce the occurrence of WMSDs and developing safe working environment. Therefore, the study aimed to assess the prevalence and associated factors of WMSD among weavers working in Bahir Dar City.

## Methods

### Study design and setting

A multi-center institutional-based cross-sectional study was conducted from April to May 2023. The study was conducted in Bahir Dar City, Ethiopia. Bahir Dar is the capital city of Amhara regional state located in the Northwestern part of Ethiopia, at a distance of 565 km far from Addis Ababa. According to the Ethiopia Central Statistics Agency, the total population of the city is ∼455,901 people in 2022 [42]. Its astronomical location is 11º35’ North latitude, 37º23’ East longitude, and 1,799 m/5,902ft above sea level [43]. In Bahir Dar City, weavers are distributed within an established institution. According to the information obtained from the Bahir Dar City Bureau of Labor and Social Affairs, more than 960 weavers have been working for more than one year in eleven [11] institutions.

### Source population

All weavers working in Bahir Dar city.

### Study Population

All eligible weavers working at 11(eleven) institutions in Bahir Dar City during the study period.

### Eligibility criteria

#### Inclusion criteria

Weavers who were 18 years old and above, working in an institution, and had more than 12 months of working experience were included in the study.

#### Exclusion criteria

Weavers who had a history of MSD before joining weaving, any trauma, and surgery at any parts of their body in the last six months before data collection were excluded from the study.

### Sample size determination

Single population proportion formulas were used to determine the sample size using the following assumptions, 95% confidence interval, 5% margin of error, and 50% proportion of WMSDs due to limited similar studies in Ethiopia and Africa.

$${\text{n = (za/2}}{{\text{)}}^{\text{2}}}\left( {\text{p}} \right)\left( {{\text{1 - p}}} \right)$$

$${d}^{2}$$

Where; n = number of sample size.

Z = 95% confidence interval i.e., 1.96.

*p* = 0.5 (proportion of WMSDs among weavers which is taken as 50%).

q = is 1- 0.5 i.e. (0.5)

d = margin of error (0.05).

Then by adding a 10% non-response rate, the final sample size of the study was 424.

### Sampling procedure and technique

**Fig. 1:**
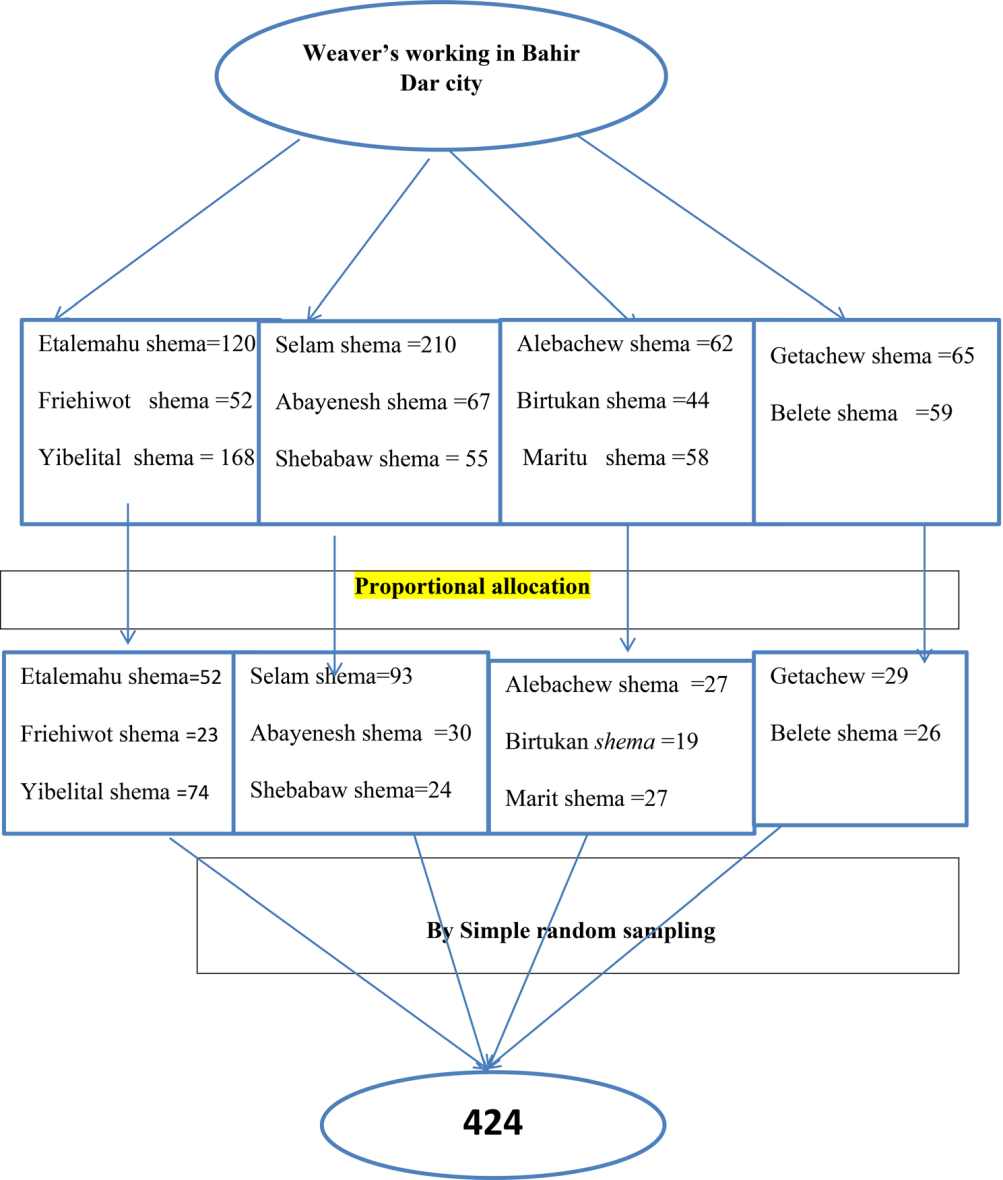
Schematic presentation showing sampling procedure and techniques among weavers working in Bahir Dar City, Northwest Ethiopia 2023

Information from Bahir Dar City Bureau of Labor and Social Affairs showed that eleven [11] weavers’ institutions were present and weavers in the city were working under a recognized institution. All institutions were included in the study and sampling frames were made following participants’ eligibility for the study. Finally, the sample size was proportionally allocated and participants were recruited using a simple random sampling technique (lottery method) (See Fig. 1).

### Study variables

The dependent variable was work-related musculoskeletal disorders responded (yes/no).

Independent variables includes; - Sociodemographic factors age, sex, BMI, educational status, and monthly family income. Lifestyle and psychosocial factors were alcohol consumption, cigarette smoking, physical exercise, job stress, and job satisfaction. In addition work-related factors were work experience, working hours, working days per week, repetitive movement, awkward posture, static posture, lack of backrest, and break time taken while working.

### Operational definitions

In this study, work-related musculoskeletal disorders are defined as aches, pain, or discomfort in any area of the body such as the neck, shoulder, upper back, elbows, lower back, wrist/hand, hips/thighs/, knee, ankle/feet at any time in the previous 12 months related to their occupation [44].

#### Awkward posture

Positions of the body that deviate significantly from the neutral position like bending or twisting while performing work activities [45].

#### Static posture

Maintaining one body alignment like sitting or standing for two hours or more without changing positions [46].

#### Repetitive movement

Weavers who had repetitive similar motions with little or no variation every few seconds for two or more hours [46].

#### Body mass index (BMI)

Measured weight in kilograms divided by the square of the height in meters (kg/m2). Underweight (< 18.50), Normal (18.50-24.99), Overweight (25–29.9), and Obesity (≥ 30) kg/m2) [47].

#### Smoker

Individuals who reported smoking daily (at least one cigarette per day) or occasionally (less than one cigarette per day) are considered smokers [48].

#### Alcoholic

A person who drinks beer, local beer, Areke, Tella, or Tej every day or every other day is considered an alcoholic [49].

#### Job stress

One’s life is appraised as stressful when the degree of perceived stress scale scores between 14 and 45 points and No = score of 8–13 points [50].

#### Job satisfaction scale

A scale used to determine the overall level of job satisfaction having ten items and a 5-point Likert-type scale where 1 = very dissatisfied, 2 = unsatisfied, 3 = neutral, 4 = satisfied, and 5 = very satisfied). Thus, Finally, workers were considered as satisfied with their jobs when the overall job satisfaction score was between 32 and 50, and “no satisfied” if they scored 10–31 on the demarcation threshold [51].

#### Physical activity

Defined as any body movement produced by skeletal muscles that requires energy expenditure of at least 30 min per day three days per week of moderate to vigorous-intensity physical activity [52].

### Data collection tool and procedures

To gather relevant data, a structured interview questionnaire adopted from different literature and a Nordic musculoskeletal questionnaire [44] were employed which include; Section I: socio-demographic characteristics (age, gender, level of education, and BMI), Section II: Lifestyle and psychosocial characteristics (smoking, alcohol drinking, physical activity, job stress, job satisfaction) and Section III: working-related factors (work experience, working hours, working days per week, repetitive movement, awkward posture, static posture, lack of backrest, and break time taken while working).

Four BSc physiotherapists data collectors and two MSc physiotherapists supervised the data collection. Before data collection, the data collectors were trained for two days about the data collection tool, how to approach study participants, the study’s purpose, and ethical considerations. A letter of request to conduct the study was prepared. Then pretest was done among 5% (20 weavers) of the sample size in Gondar town and the necessary changes were carried out on the data collection tool for the actual study. Finally, the data collector explains to the respondents the importance of their response to the study and to answer the questions with honesty.

### Data quality control

To ensure consistency, the questionnaire was first prepared in English and translated into the local language Amharic, and then back into English by experts in both languages. Four data collectors and two supervisors were trained for two days about the data collection tool, how to approach participants, and conduct interviews to have a common understanding and ensure consistency and data quality before the actual data collection. The questionnaire was pretested among Gondar town weavers. The supervisors evaluated the completed questionnaires on each day of data collection. After double-checking for consistency and completeness, the supervisors forwarded the completed surveys to the principal investigator. Those that were incomplete or missing were returned to data collectors for rectification. Supervisors rechecked 5% of the samples to see if the interviewers did well.

### Data analysis

The collected data were coded, cleaned, and entered into EPI data version 4.6 and then exported to SPSS version 25 packages for analysis. Descriptive statistics, frequency, percentages, mean, standard deviations, and logistic regression were used to describe the findings. Multi-collinearity of the independent variables was checked by variance inflation factor (VIF) cutoff point < 10. Model fitness was checked by the Hosmer- Lemeshow test > 0.005. Variables with a P-value of < 0.25 in the bivariate logistic regression analysis were accepted as potential candidates in the final multivariable logistic regression analysis. AOR and 95% confidence intervals were employed to estimate and evaluate the predictors of WMSDs included in the multivariable logistic regression. In the final model, a P-value of < 0.05 and a 95% CI are considered statistically significant.

## Result

### Socio-demographic characteristics of study participants

A total of 424 weavers were included in this study, with a response rate of 97.6% (414). From a total of 414 weavers who participated in this study, 209 (50.5%) were male, and 367 (86.6%) participants age were less than 35 years old. The mean age of the participants was 26.78 ± (6.93) and the mean BMI was 20.3 ± (2.1), In addition, the mean monthly income was 5345.4 ± (2418.3) ETB. Of the respondents, most of them 337(81.4%) had a normal body mass index. Furthermore, 212 (51.2%) participants were above grade nine in their educational status. (See Table 1).

**Table 1:** Socio-demographic characteristics of weavers working in Bahir Dar city, Northwest Ethiopia, 2023 (*n* = 414)

Variable	Frequency(*n*)	Percent (%)
**Sex**		
Male	209	50.5
Female	205	49.5
**Age group**		
< 35	367	86.6
≥ 35	47	11.4
**BMI (**kg/m2)		
Normal	337	81.4
Underweight	57	13.8
Overweight	20	4.8
**Educational status**		
Unable to write and read	49	11.8
Able to write and read	36	8.7
Grade 1-8th	117	28.3
> 9 grades	212	51.2
**monthly income (ETB)**		
≤ 5000	276	66.7
5000 − 1000	110	26.6
≥ 10,000	28	6.6

### Work-related characteristics of study participants

Among study participants, most of them 315 (72.9%) had less than five years of working experience and 256 (61.8%) were working more than eight hours per day. Regarding the number of working days, most of them 299 (72.2%) were working five and six days per week. Almost all participants had break time. Furthermore, 278 (67.1%) of participants had repetitive movements during their work. Regarding the ergonomics of the respondents, 274 (66.2%) of them had an awkward posture, and 302 (72.9%) weavers were not working in a static posture. (See Table 2).

**Table 2:** Work-related characteristics of weavers working in Bahir Dar city, Northwest Ethiopia, 2023 (*n* = 414)

Variables	Frequency (*n*)	Percent (%)
**Job experience(yrs.)**		
≤ 5	315	76.1
> 5	99	23.9
**Working hours (hrs.)**		
≤ 8	158	38.2
> 8	256	61.8
**Working days**		
3 and 4	115	27.8
5 and 6	299	72.2
**Static posture**		
No	302	72.9
Yes	112	27.1
**Repetitive movement**		
No	136	32.9
Yes	278	67.1
**Back support**		
No	132	31.9
Yes	282	68.1
**Awkward posture**		
No	140	33.8
Yes	274	66.2
**Break time**		
No	4	1
Yes	410	99

### Lifestyle and psychosocial characteristics of study participants

Regarding the lifestyle of the participants, all are nonsmokers 414 (100%), and most of them 318 (76.8%) were nonalcoholic. In addition, most of the participants 389 (94%) had no habit of doing physical exercises. Of the participants, 229 (55.3%) were satisfied with their job and 283 (68.4%) participants had job stress. (See Table 3).

**Table 3:** Lifestyle and psychosocial characteristics of weavers working in Bahir Dar city, North West Ethiopia, 2023 (*n* = 414)

Variables	Frequency (*n*)	Percent (%)
**Alcohol**		
No	318	76.8
Yes	96	23.2
**Physical activity**		
No	389	94
Yes	25	6
**Job satisfaction**		
No	185	44.7
Yes	229	55.3
**Job stress**		
No	131	31.6
Yes	283	68.4

### Prevalence of work-related musculoskeletal disorders among weavers

**Fig. 2:**
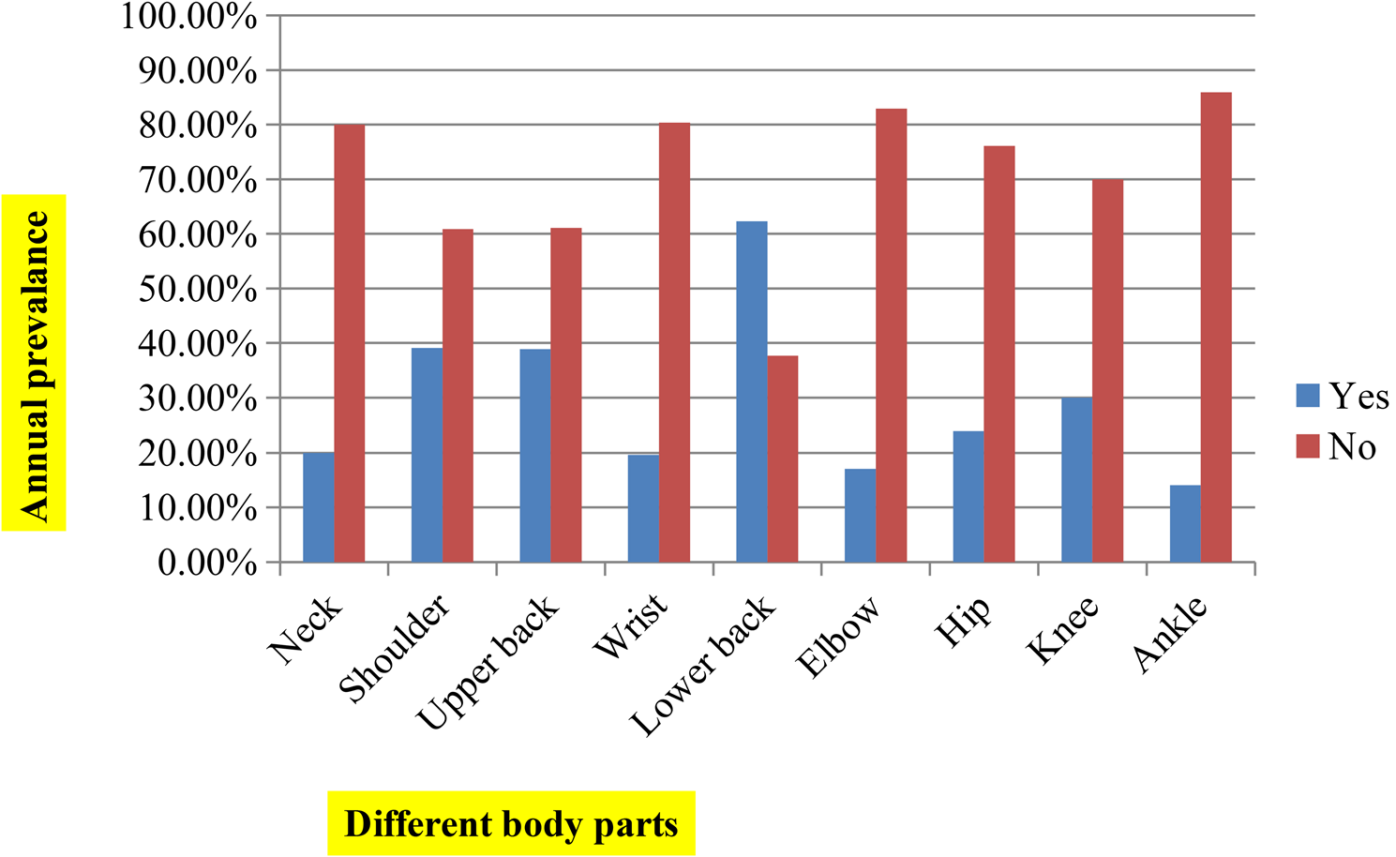
Distribution of annual prevalence of WMSDs in the nine body regions (*n* = 414)

Out of 414 weavers, the annual prevalence rate of WMSDs among weavers was 316 (76.3%) (CI = 72.2 to 80.2%). The prevalence rate during the last twelve-month period was highest in the lower back (62.3%), followed by the shoulder (39.1%) and upper back (38.9%) but less common in the elbow (17.1%) and ankle (14.7%) (See Fig. 2).

Among male participants, 163 (77.9%) of them had WMSDs and 153 (74.6%) of female participants suffered WMSDs. Out of the study participants who were developing WMSDs, 40 (85.1%) were aged greater than 35 years and 276 (75.5%) were aged less than 35. Two hundred twenty-one (86.4%) of individuals who worked more than eight hours per day had WMSDs, while 95 (62.2%) of those who worked less than eight hours per day had WMSDs. Among participants who had repetitive movement during weaving, 238(85.6%) of them had WMSDs. (See Table 4).

**Table 4:** Prevalence of WMSD among different characteristics of the participants working in Bahir Dar city, Northwest Ethiopia, 2023

Variables	Annual prevalence of WMSDs			
	No		Yes	
	Frequency (*n*)	Percent (%)	Frequency (*n*)	Percent (%)
**Sex**				
Male	46	22.1	163	77.9
Female	52	25.4	153	74.6
**Age group**				
< 35	91	24.7	276	75.3
≥ 35	7	14.8	40	85.1
**BMI (**kg/m2)				
Normal	79	23.4	258	76.4
Underweight	13	22.8	44	77.2
Overweight	6	30	14	70
**Educational status**				
Unable write and read	12	24.5	37	75.5
Able to write and read	7	19.4	29	80.6
Grade 1–8	31	26.6	86	73.5
Above grade 9	48	22.6	164	77.5
**Monthly income (ETB)**				
≤ 5000	67	24.3	209	75.7
5000 − 1000	25	22.7	85	77.3
≥ 10,000	6	21.4	22	78.6
**Working experience (yrs.)**				
≤ 5	80	25.4	235	74.6
> 5	18	18.1	81	81.9
**Working hours per day (hrs.)**				
≤ 8 h.	63	39.8	95	60.2
≥ 8 h.	35	13.6	221	86.4
**Working days per week**				
3 and 4 days	47	40.9	68	59.1
5 and 6 days	51	17.1	248	82.9
**Static posture**				
No	77	25.5	225	74.5
Yes	21	18.7	91	81.3
**Repetitive movement**				
No	58	42.6	78	57.4
Yes	40	14.4	238	85.6
**Back support**				
No	25	18.9	107	81.1
Yes	73	25.9	209	74.1
**Awake ward posture**				
No	60	42.9	80	57.1
Yes	38	13.9	236	86.1
**Alcohol**				
No	79	24.8	239	75.2
Yes	19	19.8	77	80.2
**Physical activity**				
No	90	23.1	299	76.9
Yes	8	32	17	68
**Job satisfaction**				
No	43	23.2	142	76.8
Yes	55	24	174	76
**Job stress**				
No	52	39.6	79	60.4
Yes	46	16.3	237	83.7

**Table 5:** Bivariate and multivariable logistic regression analysis of WMSDs among weavers working in Bahir Dar city, Northwest Ethiopia, 2023 (n=414)

Variable	WRMSDs		Bivariate	Multivariable	
	No(*n*)	Yes(*n*)	COR (95% CI)	AOR (95% CI)	*P* value
**Age**					
< 35	91	276	1	1	
≥ 35	7	40	1.88 (0.82–4.35)	1.35(0.49–3.70)	0.560
**Working experience**					
≤ 5	80	235	1	1	
> 5	18	81	1.532(07-2.71)	1.47(0.73–2.99)	0.280
**Static posture**					
No	77	225	1	1	
Yes	21	91	1.48 (0.86–2.55)	1.10(0.79–2.49)	0.110
**Back support**					
No	25	107	1.49(0.89–2.49)	2.50(1.29–4.48) *	0.006
Yes	73	209	1	1	
**Working hours**					
≤ 8	63	95	1	1	
> 8	35	221	4.18(2.59–6.75)	3.48(1.96–6.21) **	< 0.001
**Working days per week**					
3 and 4	47	68	1	1	
5 and 6	51	248	3.36(2.08–5.42)	1.04(0.53–2.04)	0.908
**Repetitive movement**					
No	58	78	1	1	
Yes	40	238	4.42(2.76–7.13)	4.11(2.03–8.32) **	< 0.001
**Awkward posture**					
No	60	80	1	1	
Yes	38	236	4.65(2.88–7.52)	3.37(1.87–6.06) *	0.020
**Job stress**					
No	52	79	1	1	
Yes	46	237	3.39(2.12–5.43)	4.37(2.45–7.82) **	< 0.001

*Keys* *=significant at bivariate), **=statistically significant multivariable, CI = Confidence interval, COR = crude odds ratio, AOR = adjusted odds ratio, 1 = references

### Associated factors of work-related musculoskeletal disorder among weavers

Bivariate and multivariate logistic regression models were used to analyze determinant factors. In the bivariate logistic regression analysis, age, working experience, working hours per day, repetitive movement, awkward posture, job stress, static posture, back support, and number of working days per week were associated. Finally five [5] variables remained statistically significant factors in multivariable logistic regression including working hours per day, lack of back support, repetitive movement, awkward posture, and job stress as shown in Table 5 below.

8 8Weavers Working more than eight(8) hours per day had 3.48 times higher odds of developing WMSDs than weavers working less than 8 h. [AOR = 3.48, CI = 1.96–6.21]. In addition, weavers who didn’t use back support in their workplace had 2.50 times higher odds of WMSDs than weavers who used back support [AOR = 2.50, CI = 1.29–4.48]. Weavers who had a repetitive movement for more than two hours during weaving had 4.11 times higher odds of developing WMSD than those who had less than two hours of repetitive movement [AOR = 4.11, CI= (2.029–8.324]. Furthermore, weavers who had attained an awkward posture during weaving had 3.37 times higher odds of developing WMSD than those who did not attain an awkward posture [AOR = 3.37 CI = 1.875–6.059].  Weavers those who had job stress were 4.37 times higher odds of developing WMSD compared with those who hadn’t job stress [AOR = 4.37, CI = 2.447–7.816].

## Discussion

The purpose of this study was to assess the prevalence and associated factors of WMSDs among weavers working in Bahir Dar City, Ethiopia. The current study found that the annual prevalence rate of WMSDs among weavers was 76.3% (CI = 72.2–80.2%). The annual prevalence rate of WMSD was more common in the lower back (62.3%), shoulder (39.1%), and upper back (38.9%) but less common in the elbow (17.1%) and ankle (14.7%).

The present study findings are consistent with previous studies conducted in India (75.9%) and Indonesia (80.5%) [27, 38]. This could be attributed to comparable study participants in terms of educational status, BMI, and work experiences, as well as the use of Nordic musculoskeletal questionnaires in data collection. For instance, in an Indian study, 75.5% of individuals had a normal BMI. Similarly, more than two-thirds of participants in our study had a normal BMI. On the other hand, the majority of participants in the Indonesian had less than 5 years of job experience. Likewise, the majority of participants in our study had less than 5 years of job experience.

The current study finding was lower than the studies in India (100%) and Bangladesh (100%) [32, 37]. This variation might be due to those studies having relatively older age participants, different sampling techniques (convenient sampling), longer working experiences, lower educational levels, and long working hours. The studies done in India and Bangladesh mean ages of study participants was 54.65 ± (3.15) and 39.48 (± 7.78) years respectively, while in our study the mean age of the participants was 26.78± (6.93). Evidence also showed that an increase in age leads to higher episodes of WMSD [53]. Furthermore, In studies done in India 44.4% of the participants had no formal education and more than half of the participants in Bangladesh were illiterate but in the current study, more than half of the participants were above grade nine educational status. This could be because those who have low educational levels may have low knowledge and skills about workplace ergonomics. Furthermore, the study participants in Bangladesh were selected by using a convenient sampling technique, which may have resulted the data not truly representative of the entire population as a whole. In addition, the majority of participants in our study had less than five years of work experience, both studious indicated that all participants had more than five and ten years of work experience. This might be in comparison to a short length of work experience, prolonged job experience may have been sufficiently exposed to risk factors that may cause cumulative trauma or recurrent strains that arise gradually from overuse leads to work-related musculoskeletal disorders. In contrast to our study, participants in Bangladesh had more than 8 h of working, whereas in our study, more than one-third of the weavers had less than eight hours working time. This might be becauseof working longer hours can lead to WMSDs [54].

In addition, The current study finding was lower than the study done in Bangladesh (82.4%) [19]. This disparity resulted from the participants’ lower levels of education and advanced age. As an instance of variation, study conducted in Bangladesh, more than half of the participants were older than 40, but in our study, more than two-thirds of the participants were younger than 35. As a person gets older their resilience to WMSDs decreases and a person’s tissue strength deteriorates, which increases the severity and frequency of soft tissue injury [55]. In the study done in Bangladesh, greater than half of participants were illiterate but in the current study, more than half were above grade nine. This might be because those who had a low educational status may have less knowledge and skills in the prevention of WMSDs.

The current study reported that higher prevalence of WMSD than the study done in Bangladesh (27.4%) [13]. This variation may result from the use of different data collection tools and sex of participants. In the study done in Bangladesh Participants were exclusively male While nearly half of the participants in the current study were female. This might be men are less likely to develop WMSD than women because of variations in body size, muscle mass, hormone levels, and work-life balance [56]. Another difference might be because the Nordic Musculoskeletal Questionnaire was used to collect data for our study, whereas components of a standardized Cultural and Psychosocial Influences on Disability (CUPID) were used to collect data for the study conducted in Bangladesh. This could account for a discrepancy in the tool’s sensitivity and specificity.

According to the findings of the present study long working hours, lack of back support, repetitive movement, awkward posture, and job stress were significantly associated with WMSDs among weavers working in Bahir Dar city.

Our study revealed that weavers who worked more than eight hours a day had 3.48 times higher odds of developing WMSD than weavers working less than eight hours. . The reason could be that working more hours per day can result in a relative reduction in the amount of time needed to recover from accumulated fatigue and relax after stressful situations. These factors will eventually have an impact on the musculoskeletal system and may ultimately lead to WMSDs [54]. This study is supported by studies done in Bangladesh [13, 32] and Indonesia [38].

In addition, weavers who did not have back support were 2.50 times more likely to have WMSD than those having back support. This might be due to the negative effect of the adopted posture during the weaving operation. These might be back support would be required for back muscle support during rest pauses that reduces intra-discal pressure and eventually reduces stress on spinal and paraspinal structures [57, 58]. This study is supported by the study done in Ethiopia [33] and study conducted in Belgium on female textile workers sitting postures without back support [59].

Moreover, weavers having repetitive movement during weaving were 4.11 times more likely to develop WMSD than those who didn’t have repetitive movement for two or more hours at a time. This could be explained that the amount of work repetitions that strain the musculoskeletal system, which raises the risk of exhaustion and insufficient time for tissue healing, which results in pain and discomfort [36, 60]. This study is supported by the study done in India [23].

Furthermore,  weavers who had an awkward posture are 3.37 times more likely to develop WMSD than who did not attain awkward posture . This could bethe fact that the body’s posture affects the amount of force generated by the muscles and joints when performing an activity. Bending or twisting the body puts more strain on the joints, muscles, and nerves, which can wear them out and result an injuries [61]. This study is consistent with the study done in India [62]. Likewise, Another study done in India [23] and Bangladesh [32] supported our study.

In terms of psychosocial factors, those who had job stress during work were 4.37 times more likely to develop WMSD than those who didn’t have job stress. This might be stress induces physiological alterations in humans, mostly affecting the neurological and endocrine systems. Excessive and prolonged stress causes musculoskeletal diseases by tightening muscles and reducing micropauses in muscle activity [63]. This study finding is supported by a study done in Bangladesh [13] and studies done among bank workers in Ethiopia and Kuwait [64, 65]. Generally, the findings of this study provide good insight into health promotion, reduce the occurrence of WMSD, and develop the intervention plan and prevention of factors that induce WMSD in weaving operation workers.

## Limitations of the study

The main limitation of this study was recall bias due to data being collected based on a self-report. .   Another limitation was because of the scope of our study was restricted to Weaver’s institution, its conclusions cannot be extended to the community as a whole. Apart from its limitations, our study is the first study evaluating the prevalence and associated factors of WMSD among weavers in Ethiopia and will add good insights into the existing knowledge in the area.

## Conclusion and recommendation

The findings of our study indicated a high prevalence of WMSDs among weavers. Longer working hours per day, lack of back support, repetitive movement, awkward posture, and job stress were significantly associated with WMSDs. We recommended that all stakeholders focus on the prevention measures to reduce the burden of WMSDs. In addition, weavers and employers have to focus on avoiding awkward posture, using back support while sitting, avoiding/minimizing repetitive movement during weaving, reducing the working hours per day (less than or equal to eight hours per day), and minimizing job-related stress. Furthermore, we would like to recommend researchers to conduct longitudinal studies with objective measurement.

## Data Availability

The data that support the findings of this study are available from the principal investigator upon reasonable request fisehaseifu44@gmail.com.

## Abbreviations

AOR: Adjusted Odds Ratio
BMI: Body Mass Index
CI: Confidence Interval
ETB: Ethiopian Birr
LBP: Lower Back Pain
MSDs: Musculoskeletal Disorders
MSP: Musculoskeletal Pain
SPSS: Statistical Package for Social Science
VIF: Variance Inflation Factor
WHO: World Health Organization
WMSD: Work-Related Musculoskeleta1 Disorders
